# Molecular characterisation of rifampicin-resistant *Mycobacterium tuberculosis* strains from Malawi

**DOI:** 10.4102/ajlm.v6i2.463

**Published:** 2017-03-31

**Authors:** Tarsizio Chikaonda, Irene Ketseoglou, Nelson Nguluwe, Robert Krysiak, Isaac Thengolose, Felix Nyakwawa, Nora E. Rosenberg, Christopher Stanley, James Mpunga, Irving F. Hoffman, Maria A. Papathanasopoulos, Mina Hosseinipour, Lesley Scott, Wendy Stevens

**Affiliations:** 1 Department of Molecular Medicine and Haematology, Faculty of Health Sciences, School of Pathology, University of the Witwatersrand, Johannesburg, South Africa; 2 UNC Project, Lilongwe, Malawi; 3 Malawi National Tuberculosis Programme, Lilongwe, Malawi; 4 University of North Carolina at Chapel Hill, Chapel Hill, North Carolina, United States

## Abstract

**Background:**

Availability and access to the detection of resistance to anti-tuberculosis drugs remains a significant challenge in Malawi due to limited diagnostic services. The Xpert^®^ MTB/RIF can detect *Mycobacterium tuberculosis* and resistance to rifampicin in a single, rapid assay. Rifampicin-resistant *M. tuberculosis* has not been well studied in Malawi.

**Objectives:**

We aimed to determine mutations in the rifampicin resistance determining region (RRDR) of the *rpo*B gene of *M. tuberculosis* strains which were defined as resistant to rifampicin by the Xpert MTB/RIF assay.

**Methods:**

Rifampicin-resistant isolates from 43 adult patients (≥ 18 years) from various districts of Malawi were characterised for mutations in the RRDR (codons 507–533) of the *rpo*B gene by DNA sequencing.

**Results:**

Mutations were found in 37/43 (86%) of the resistant isolates in codons 511, 512, 513, 516, 522, 526 and 531. The most common mutations were in codons 526 (38%), 531 (29.7%) and 516 (16.2%). Mutations were not found in 6/43 (14%) of the resistant isolates. No novel *rpo*B mutations other than those previously described were found among the rifampicin-resistant *M. tuberculosis* complex strains.

**Conclusion:**

This study is the first to characterise rifampicin resistance in Malawi. The chain-termination DNA sequencing employed in this study is a standard method for the determination of nucleotide sequences and can be used to confirm rifampicin resistance obtained using other assays, including the Xpert MTB/RIF. Further molecular cluster analysis, such as spoligotyping and DNA finger printing, is still required to determine transmission dynamics and the epidemiological link of the mutated strains.

## Introduction

Tuberculosis remains an important public health problem especially in the developing world. The global impact of tuberculosis is significant, with an annual estimate of 9.6 million tuberculosis cases and over 1.5 million deaths due to tuberculosis in 2014.^1^ The tuberculosis burden is worsened by the emergence and spread of multi-drug resistant (MDR) tuberculosis cases, defined as simultaneous resistance to at least rifampicin and isoniazid, with or without resistance to any other drug.^1^

In Malawi, there were 5564 new smear-positive cases of tuberculosis registered in 2014.^1^ Patients diagnosed with tuberculosis are treated with the standard quadruple antibiotic therapy recommended for drug-susceptible tuberculosis (rifampicin, isoniazid, ethambutol and pyrazinamide). Rapid detection of drug resistance is crucial in choosing the most effective treatment to avert morbidity and mortality of infected individuals and reduce the risk of MDR tuberculosis transmission.^1^

Rifampicin, if the isolate is susceptible, is a very important component of the current tuberculosis treatment regimen and has proved to be effective to both susceptible strains and strains resistant to streptomycin and isoniazid.^1^ However, there is growing resistance to rifampicin, largely due to particular genomic mutations in the *rpo*B gene of *Mycobacterium tuberculosis*.^2^ The *rpo*B gene encodes the *β* subunit of RNA polymerase, which is involved in chain initiation and elongation. A signature sequence for *M. tuberculosis* identification is contained in this region.^3,4^ Mutations in the rifampicin resistance determining region (RRDR) of this gene (codons 507–533) are associated with rifampicin resistance.^5^ Detection of such mutations indicates rifampicin-resistant tuberculosis strains and can be used as a predictor for MDR tuberculosis, although not a complete surrogate marker.^6^

Globally, it is estimated that 3.3% of new cases and 20% of previously-treated cases have MDR tuberculosis and that 9.7% of these cases have pre-extensively drug resistant tuberculosis.^1^ A substantial percentage (37%) of new tuberculosis cases and a staggering percentage (74%) of the global estimate of MDR tuberculosis incident cases were not reported or remained undiagnosed in 2014. Determination of the pattern of drug resistance is performed in less than 3% of people diagnosed with tuberculosis worldwide.^1,7,8^

Phenotypic drug susceptibility testing (DST) relies on detection of growth and is performed using an antibiotic susceptibility testing set consisting of a growth control and one tube for each anti-tuberculosis test drug, with known concentration.^9^ Phenotypic DST is widely used in limited-resource settings. This method is inexpensive and accurate, though time consuming due to its reliance on the growth of *M. tuberculosis,* which takes a long time to obtain results.^10^ Mutation(s) in the genes relevant to responses to each drug are associated with resistance to tuberculosis drugs.^10^ Genotypic DST methods target well-characterised resistance-associated mutations. Genotypic DST determines such mutations in the tested gene region only and as such, unknown or less frequent mutations might be missed.^11,12^ Molecular tools for rapid DST are developed upon a better understanding of mutations responsible for drug resistance in the bacterial genome.^13,14^ Several molecular methods, including the Xpert^®^ MTB/RIF (Cepheid, Sunnyvale, California, United States), have been developed for the detection of *M. tuberculosis* complex DNA and RRDR mutations associated with rifampicin resistance.^15,16,17^ The World Health Organization strongly recommends the use of Xpert MTB/RIF as the initial diagnostic test for use on pulmonary specimens from adults and children suspected of having HIV-associated tuberculosis or MDR tuberculosis.^1^

The use of DNA sequencing complements the above assay in detecting new mutations, as well as confirming the presence of the most frequent mutations that could be associated with drug resistance, and has conferred excellent benefits to patient care due to the larger DNA fragment that is sequenced.^18,19^ DNA sequencing determines mutations by comparing the differences between the gold standard (H37Rv reference strain) and the test nucleotide sequence.^20,21^

We aimed to assess the RRDR of the *rpo*B gene for mutations in *M. tuberculosis* strains that were defined as rifampicin resistant by the Xpert MTB/RIF assay and to establish the prevalence of such mutations in tuberculosis-infected Malawians.

## Methods

### Ethical considerations

Approvals for this study were granted by University of the Witwatersrand Human Research Ethics Committee (M120256), National Health Sciences Research Committee (NHSRC) in Malawi (NHSRC # 999) and the University of North Carolina (UNC; Chapel Hill) Institutional Review Board (CID 1211). Obtaining consent was waived because the study used previously-stored and residual sputum pellets, and consent from patients was given during recruitment and/or this was part of routine testing for tuberculosis drug resistance.

### Study population

We conducted this study using processed sputum sediments (pellets) from new and previously-treated patients, ≥ 18 years of age. Demographic and clinical information was collected from the respective laboratory tuberculosis registers and entered in Excel (Microsoft, Inc., Redmond, Washington, United States) spreadsheets. Both retrospective and prospective pellets (*N* = 995) were used in the current study.

### Retrospective pellets

Retrospective sputum pellets (*n* = 351) were collected between April 2011 and July 2012 from a study conducted in outpatients initiating tuberculosis treatment at Martin Preuss Centre at Bwaila Hospital (Bwaila) in Lilongwe, which was looking at the prevalence of drug-resistant tuberculosis at this HIV/tuberculosis clinic.^22^ Sputum samples were processed for routine culture at the UNC Project laboratory following the N-acetyl-L-cysteine and sodium hydroxide (NALC-NaOH) method. Pellets were inoculated on both mycobacterium growth indicator tubes (MGIT) and Löwenstein Jensen media. DST was performed using the Hain MTBDR*plus* assay (Hain Lifescience GmbH, Nehren, Germany). Residual pellets were stored at -80°C and selected at random for use in this study without any special criteria to eliminate bias.

### Prospective pellets

Prospective sputum pellets came from patients suspected of drug-resistant tuberculosis (*n* = 644) who presented consecutively to district hospitals in Malawi, and sputum samples were sent to the National Tuberculosis Reference Laboratory (NTRL) in Lilongwe for conventional DST between June 2012 and May 2014. Sputum was processed using the NALC-NaOH method and the pellet was split in two. The first sample was used for routine culture and conventional DST at the NTRL, while the other was stored for up to two weeks at 2°C – 8°C for Xpert MTB/RIF testing and MGIT culture at the UNC laboratory.

### Mycobacterium cultures at the UNC laboratory

Pellets were re-suspended in 1.5 mL of phosphate buffer and split into different volumes. One part was processed for routine culture using MGIT and the other was processed on Xpert MTB/RIF (0.5 mL). MGIT tubes were inoculated with 500 µL of the re-suspended sample as previously described^23^ and were monitored daily for growth using a hand-held BACTEC MicroMGIT Reader for up to 42 days. Smears were prepared from positive cultures, stained using Ziehl-Neelsen stain (Becton, Dickinson & Company, Sparks, Maryland, United States) and examined for acid-fast bacilli. Determination of eligibility for DNA sequencing was dependent on results from the Xpert MTB/RIF assay (software version G4). DNA was extracted from corresponding MGIT cultures if rifampicin resistance was detected by Xpert MTB/RIF.

### Xpert MTB/RIF assay

Samples (*n* = 995) were processed as per the manufacturer’s instructions. A sample reagent buffer was added to 500 µL of the re-suspended pellet in the ratio of 3:1 (sample reagent buffer:specimen) as described previously.^24^ The container was closed tightly and then vigorously shaken for 15 seconds. The mixed specimen was left to stand for 10 minutes followed by another vigorous shaking for 15 seconds and left to stand for a further five minutes. Thereafter, 2 mL of the mixed specimen was loaded into a single-use Xpert MTB/RIF cartridge. The closed cartridge was loaded into the GeneXpert^®^ instrument, where extraction, amplification and detection of *M. tuberculosis* and screening for resistance to rifampicin were performed automatically and simultaneously. Results were available within two hours. The Xpert MTB/RIF probe and cycle threshold (Ct) value was documented for each tuberculosis strain that was detected as resistant to rifampicin.

### DNA extraction and amplification

Genomic bacterial DNA was extracted from cultures at the UNC laboratory using the HAIN GenoLyse kit (Hain Lifescience GmbH, Nehren, Germany). From MGIT liquid media, 1.0 mL was transferred to a Sarstedt micro-centrifuge tube. The tube was centrifuged for 15 minutes at 14 000 rpm. The supernatant was carefully discarded and the pellet was re-suspended in 100 µL Lysis Buffer from the kit, vortexed thoroughly and incubated at 95°C in a heating block for five minutes. Tubes were removed and briefly centrifuged to remove condensation. To each tube, 100 µL of Neutralization Buffer was added, vortexed for five seconds and then centrifuged for five minutes at 14 000 rpm. Supernatant was transferred to a new tube and the pellet was discarded. The extracted DNA was stored at -80°C for use as DNA template.

### MTBDR*plus* line probe assay

The GenoType^®^MTBDR*plus* line probe assay (Version 2) was performed according to the manufacturer’s instructions. In brief, an appropriate number of PCR tubes, one for each sample and one each for a PCR positive (*M. tuberculosis* DNA) and negative (dH_2_O) control were labelled. A master mix was prepared for one reaction and the final volume adjusted according to the total number of samples, with some excess to allow for pipetting errors. The solution was mixed by inversion and an aliquot of 45 µL of the prepared master mix was transferred to each PCR tube. 5 µL of each sample or control DNA was added to the appropriate PCR tube. PCR was performed in a 9700 DNA thermocycler (Applied Biosystems, Foster City, California, United States), using the cycling protocol as described by the manufacturer.

### DNA PCR

PCR amplification of the *rpo*B gene, which included the RRDR, was carried out using forward primer *rpo*BF2 (5’-GAG GGT CAG ACC ACG ATG AC-3’; nucleotide positions 1030 to 1049 according to H37Rv numbering, GenBank accession number CAB09390.1) and reverse primer *rpo*BR (5’-GAG CCG ATC AGA CCG ATG T-3’; nucleotide positions 1460 to 1478 according to H37Rv numbering) in a GeneAmp PCR system 9700 thermocycler (Applied Biosystems, Foster City, California, United States). The total volume of the PCR reaction used was 50 µL, containing: 5 µL of 10X high fidelity buffer, 1 µL of 10 mM dNTP mix, 2 µL of 50 mM MgSO_4_, 1 µL of each primer, 0.2 µL of Taq HiFi (Invitrogen, Carlsbad, California, United States), 36.8 µL of molecular grade water and 3 µL of extracted genomic DNA. Amplification conditions were set at: 94°C for two minutes; followed by 35 cycles of 94°C for 30 seconds, 55°C for 30 seconds, 68°C for 40 seconds; followed by a 10-minute final elongation at 68°C. PCR products were purified using the GeneJet PCR purification kit (Thermo Scientific, Waltham, Massachusetts, United States) following the manufacturer’s instructions. 5 µL of the amplified product was visualised on a 1% agarose gel.

### DNA sequencing

DNA sequencing was performed in an automated DNA sequencer ABI 3700, (Applied Biosystems, Foster City, California, United States) using the BigDye terminator V3.1 sequencing kit with forward primer *rpo*BS (5’-GCA GAC GTT GAT CAA CAT CC-3’) and reverse primer *rpo*BR (5’-GAG CCG ATC AGA CCG ATG T-3’). The total volume of the sequencing reaction was 20 µL, containing: 4 µL of BigDye terminator, 2 µL of 10X sequencing buffer, 2 µL of water, 1 µL of primer and 11 µL of sample. Amplification conditions were set for: one minute at 96°C; followed by 25 cycles of 96°C for 10 seconds, 50°C for five seconds, 60°C for four minutes; followed by a 10-minute final extension at 72°C. The completed sequencing reaction mixture was purified using 75% isopropanol as per the manufacturer’s instructions.

The sample plate was loaded onto the ABI 3700 automated DNA sequencer and the resulting sequences were analysed and compared to the wild-type sequence of the well-characterised *M. tuberculosis* H37Rv reference strain using Sequencher software V4.8 (Genecodes Corporation, Ann Arbor, Michigan, United States). The well-defined RRDR region (codons 507–533) corresponded to codons 426–452 (nucleotides 1276–1356) in the downloaded H37Rv reference sequence.

### Statistical analysis

All study data were entered into an Excel spreadsheet and then exported and analysed using Stata Version 12 (StataCorp, College Station, Texas, United States). We calculated proportions and percentages based on district, patient category, Xpert MTB/RIF probes and codons in the RRDR of the *rpo*B gene to determine resistance mutations and their prevalence.

## Results

The 644 specimens from NTRL were categorised clinically as previously treated cases (574/644; 89.1%), new (27/644; 4.2%), or not known (43/644; 6.7%). From Bwaila, 24/351 (6.8%) suspected tuberculosis cases were categorised as retreatment, and 327/351 (93.2%) were from patients presenting with tuberculosis for the first time.

**FIGURE 1:**
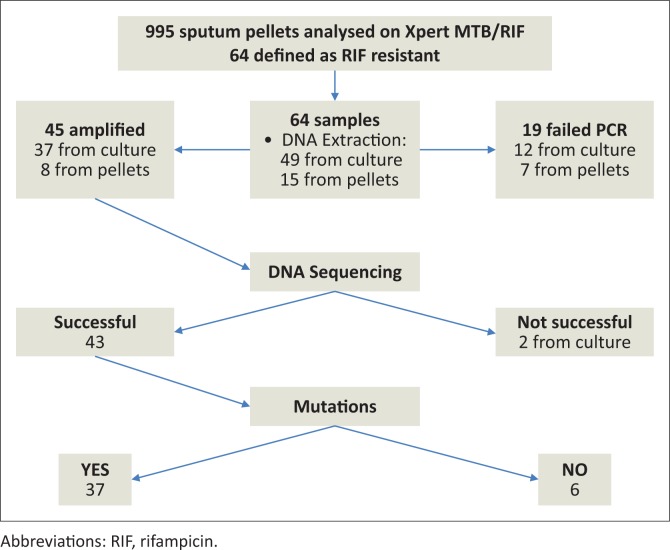
Workflow and isolate selection for DNA sequencing from tuberculosis cultures and processed sediments.

The Xpert MTB/RIF assay detected rifampicin resistance in 64/995 (6.4%) specimens, which were selected for further DNA sequencing of the larger region of the *rpo*B gene. Three specimens (3/64) were from Bwaila, while the rest (61/64) were collected from NTRL. Most of the rifampicin-resistant cases detected by Xpert MTB/RIF were associated with probe B (23/64) and probe E (23/64). Fifteen (15/64) samples were detected by probe D, 2/64 by probe A, while probe C detected 1/64 samples (Table 1). Of these, 43/64 were successfully sequenced. The remainder (*n*= 21; 32.8%), all from NTRL, were not sequenced, either due to failure of PCR amplification (*n* = 19) or they failed sequencing (*n* = 2) (Figure 1). The PCR amplification failure could not be explained, although in part this could be due to insufficient template DNA, a an attempt was made to extract DNA from pellets (*n* = 7) which had not grown on culture.

**TABLE 1:** *rpo*B gene mutations associated with Xpert MTB/RIF probes used.

DNA sequencing mutations	Xpert MTB/RIF probes					Total
	A	B	C	D	E	
D516V	1	4	0	0	1	6
Failed sequencing	0	2	0	0	0	2
H526D	0	0	0	1	0	1
H526R	0	0	0	2	0	2
H526Y	0	1	0	10	0	11
L511P	1	0	0	0	0	1
Wild-type	0	4	0	0	2	6
Failed PCR amplification	0	10	0	2	7	19
Q513E	0	1	0	0	1	2
S512T	0	0	0	0	1	1
S513L	0	0	0	0	1	1
S522L	0	0	1	0	0	1
S531L	0	1	0	0	9	10
S531Y	0	0	0	0	1	1
**Total**	**2**	**23**	**1**	**15**	**23**	**64**

Seven different mutations were detected in 37/43 (86%) specimens in codons 511, 512, 513, 516, 522, 526 and 531. Mutations were common in codons 526 (38%), 531 (29.7%) and 516 (16.2%) (Table 2). No insertion, deletion or double point mutations were observed. However, mutations were not detected in 6/43 (14%) of the strains by DNA sequencing, despite being detected by Xpert MTB/RIF probe B (4/6) and by probe E (2/6) as rifampicin resistant. Repeat Xpert MTB/RIF testing was not done for these strains due to insufficient left-over pellets. Delayed Ct values between 17.0 and 32.7 were observed in 4/6 strains which had no mutations. The observed Ct values were markedly higher than the ΔCt max cutoff of > 4 for the automated detection of rifampicin resistance by the Xpert MTB/RIF assay.^25^ The remainder (*n* = 2) gave an undetectable result on probe B (*n* = 1) and probe E (*n* = 1).

**TABLE 2:** Distribution of mutations in codons 491–574 of the *rpo*B gene and amino acid changes in the rifampicin resistance determining region, Malawi, 2011 and 2014†.

Mutated codon	Amino acid change		Nucleotide change		Total isolates (*n* = 37)	Percentage
	From	To	From	To		
511	Leu	Pro	CTG	CCG	1	2.7%
512	Ser	Thr	AGC	ACC	1	2.7%
513	Gln	Leu	CAA	CTA	1	8.0%
		Glu	CAA	GAG	2	
516	Asp	Val	GAC	GTC	6	16.2%
522	Ser	Leu	TCG	TTG	1	2.7 %
526	His	Arg	CAC	CGC	2	38%
		Tyr		TAC	11	
		Asp		GAC	1	
531	Ser	Leu	TCG	TTG	10	29.7%
		Tyr		TAG	1	

† DNA sequencing results using sputum pellets and culture specimens of 37 tuberculosis-infected patients.

When stratified by district, the prevalence of mutations was high in tuberculosis patients from Ntchisi 2/5 (40%), Nkhotakota 4/13 (30.8%), Nsanje 5/20 (25%) and Balaka 2/12 (16.7%). Lilongwe, the capital city of Malawi, had the lowest prevalence of mutations 9/466 (1.9%), followed by Mulanje 2/57 (3.5%).

As expected, the number of mutations was highest among patients registered as retreatment 29/598 (4.8%), compared with new cases 8/354 (2.3%). There were no real trends of mutations across classified retreatment cases (recurrent, default, etc.) in relation to new tuberculosis cases. Mutations in codon 531 were found in almost all patient groups, except for those classified as recurrent; codon 526 in all groups, except in the ‘new patient’ category in which mutations at codon 516 were predominant. A single strain from a patient with no history of prior tuberculosis treatment was detected with a mutation at codon 522.

All 43 specimens that were successfully sequenced were also tested on GenoType MTBDR*plus* and two strains gave discordant results. Both strains showed susceptibility to rifampicin on GenoType MTBDR*plus*, but resistance to rifampicin on Xpert MTB/RIF. Mutations were not detected in these strains by DNA sequencing.

## Discussion

This study is the first to characterise rifampicin resistance in Malawi. The majority of *rpo*B gene mutations in this study analysed by direct sequencing correlated well with rifampicin resistance observed on Xpert MTB/RIF. Our findings reveal that 86% of all rifampicin-resistant isolates harboured mutations in the RRDR of the *rpo*B gene with codon 526 (CAC → TAC) as the most frequent, followed by codon 531 (TCG → TTG/TAG). By contrast, mutations were not detected in 6/43 (14%) of the strains following nucleotide sequencing. False-positive rifampicin resistance was most likely considering the observed wild-type sequences in these strains. Data on drug-resistance mutations involving the RRDR of the *rpo*B gene, which relates highly to rifampicin resistance, is new for Malawi as no previous studies have analysed the *rpo*B gene among tuberculosis strains circulating in the country.

Previous reports demonstrated that strains requiring high rifampicin concentrations in phenotypic DST have been associated with mutations at codons 526, 516, and 531,^26,27^ corroborating that these are the most prevalent *rpo*B mutations worldwide.^28^ Several studies of rifampicin-resistant tuberculosis have reported the presence of novel and common *rpo*B gene mutations, especially in the RRDR.^29,30,31^ In addition, other studies have documented the presence of common and novel *rpo*B mutations outside the RRDR.^29,32^ Based on our amplicon size, we only evaluated the most frequently-mutated codons between positions 491–574, which include the RRDR. The contribution of additional mutations to resistance at positions outside the region that was sequenced, for example codon 176, cannot be ruled out. Future work should focus on sequencing the entire *rpo*B gene.

It is of particular interest that the Xpert MTB/RIF assay detected rifampicin resistance in six isolates, but partial sequencing of the *rpo*B gene did not detect any changes in the nucleotide sequences, such that the mechanisms/pathways of resistance of these strains remain unknown. Since unprocessed sputum was not sequenced, it was difficult to confidently exclude hetero-resistance in these strains. In low rifampicin-resistance prevalence areas, the Xpert MTB/RIF assay would be expected to falsely diagnose some cases,^8^ which could be the case with these strains. Sequencing the entire *rpo*B gene might help to understand the mechanism of resistance of the strains. In contrast, results obtained in Swaziland show that the Xpert MTB/RIF assay did not detect the *rpo*B I491F mutation (outbreak strain) in 38/125 (30%) of the isolates tested as compared to DNA sequencing, raising fears about the assay’s unreliability due to under-diagnosis and potential treatment inadequacy, since it does not detect mutations outside the RRDR.^33^ Furthermore, a study by Theron et al. observed that five strains were rifampicin resistant on Xpert MTB/RIF, *rpo*B sequencing and/or GenoType MTBDR*plus*, but susceptible on phenotypic DST.^34^ It should be noted, however, that PCR amplicons were different in the Xpert MTB/RIF assay versus what was used for the Sanger-based sequencing. The probes may have bound to minority variants in the Xpert MTB/RIF amplicons, resulting in a positive signal, whereas a combination of PCR bias and population-based Sanger sequencing (where the limit of detection is approximately 20% of the quasispecies and only the predominant population is reported) might have resulted in only the wild-type sequence being detected.^35^ Moreover, different fitness of drug-resistant tuberculosis under culture conditions can allow for the outgrowth of wild-type tuberculosis,^36^ accounting for the discordant results between the molecular and phenotypic assays.

Drug resistance was detected in some new tuberculosis cases and these results were confirmed by detecting mutations in the RRDR of the *rpo*B gene. Detecting resistance to rifampicin in new tuberculosis cases highlights the need for testing resistance to drugs in this category especially if the patient was in contact with a known MDR tuberculosis patient. Our results show that the prevalence of mutations associated with rifampicin resistance among new tuberculosis cases was 2.3%. In Malawi, the prevalence of MDR tuberculosis is low (0.4%) among new smear-positive cases and is 4.8% among retreatment cases. Testing for tuberculosis drug resistance in Malawi is not routinely done except for those highly suspected of drug resistance.^37^

Results obtained by the Xpert MTB/RIF assay were in agreement with GenoType MTBDR*plus* results on rifampicin resistance, with an exception of 2/43 (4.7%), which were sensitive on the GenoType MTBDR*plus* but rifampicin resistant on Xpert MTB/RIF. Ct values of 18.5 and 24.4 on Xpert MTB/RIF, both on probe B, were interpreted as rifampicin resistant. Mixed infection with multiple tuberculosis strains was excluded in these strains given the wild-type *rpo*B gene sequences and no observed underlying peaks on the DNA sequence chromatograms. Phenotypic DST is difficult to standardise and expensive and time-consuming to maintain routinely. In the absence of molecular techniques, several critical questions about tuberculosis remain unanswered, including recognition of acquisition of drug resistance resulting from gene mutations versus transmission of drug resistant strains.^38^

### Limitations

The relatively small number of rifampicin-resistant samples observed in the current study limited our ability to establish the overall prevalence of *rpoB* gene mutations in tuberculosis strains circulating in Malawi. Moreover, we sequenced only an approximately 450 bp amplicon; thus, the contribution of mutations in regions outside of the amplicon sequenced to overall resistance could not be determined. With the available data, an assessment of the epidemiological link between the rifampicin-resistant strains was not possible, as such further molecular cluster analysis is still required to determine transmission dynamics of the mutated strains. Future studies should consider performing phenotypic DST to detect mutations outside the RRDR (full length *rpo*B gene) and compare to DNA sequencing, considering that Sanger sequencing has a limit of detection where the minority population may not be detected.

### Conclusion

The chain termination DNA sequencing employed in this study is a standard method for the determination of nucleotide sequences. It is conclusive and can be used to confirm rifampicin resistance obtained by other assays, including the Xpert MTB/RIF assay, despite having a limit of detection with minority resistant populations. Although phenotypic DST is recommended to verify rifampicin resistance when using Xpert MTB/RIF, DNA sequencing can be used to detect the frequency and exact site of mutations in the RRDR. All tuberculosis strains with mutations in the current study had one of the previously-described *rpo*B gene mutations containing nucleotide changes. No new mutations were identified. Findings from this study emphasise the need for a national representation of sputum specimens from tuberculosis-infected patients to assess the magnitude of tuberculosis *rpo*B gene mutations that are responsible for rifampicin resistance in Malawi.
